# Planimetric quantification of plaque in patients with multibracket appliances using an intraoral scanner – proof-of-concept

**DOI:** 10.1007/s00784-026-06809-8

**Published:** 2026-03-18

**Authors:** Katja Jung, Carolina Ganss, Heike Korbmacher-Steiner, Anahita Jablonski-Momeni

**Affiliations:** 1 Marburg University, University Dental Medicine, Clinic for Operative Dentistry, Endodontics, and Pediatric Dentistry, Section for Cariology, Marburg, Germany; 2 Marburg University, University Dental Medicine, Department of Orthodontics, Marburg, Germany

**Keywords:** Orthodontics, Fixed multibracket (MB) appliances, Oral hygiene, Planimetry, Intraoral scanner (IOS)

## Abstract

**Objectives:**

This proof-of-concept study aimed to evaluate whether disclosed dental plaque can be reliably visualized and quantified using an intraoral scanner in patients with multibracket (MB) appliances, despite potential imaging artefacts caused by metallic brackets.

**Materials and methods:**

Twenty patients (mean age: 17.0 ± 2.1 years) with fixed MB (Mini Sprint^®^ II or SPEED brackets) underwent 3D-intraoral scans before (T1) and after (T2) bracket debonding. Plaque was visualized using a disclosing agent and quantified planimetrically on selected Ramfjord teeth (FDI 16, 21, 24, 36, 41, 44) using standardized image processing. Five image sets per tooth were analysed to compare plaque coverage (P%) under various conditions: with brackets, after debonding, with masked bracket areas and with the masked areas transferred onto surfaces after debonding, and with only the exposed surface considered. Statistical analyses included t-tests and Bland-Altman plots.

**Results:**

Highest P% values were found with brackets in situ (49.4 ± 8.9 P%), followed by values obtained after bracket debonding (34.0 ± 7.1 P%). After masking bracket areas, plaque levels approximated those seen after debonding (29.8 ± 7.0 vs. 34.0 ± 7.1 P%; *p* < .001). No significant difference was observed between bracket types. Reliable plaque quantification was achieved when bracket areas were masked in both scan sets.

**Conclusions:**

Intraoral scans enable valid planimetric plaque quantification in patients with MB, especially when metallic bracket areas are masked during analysis.

**Clinical relevance:**

Digital intraoral scans, combined with plaque disclosing agents, offer a reproducible and objective method for assessing plaque levels in orthodontic patients. This approach may support individualized hygiene monitoring and patient education during fixed appliance therapy.

## Introduction

Fixed multibracket (MB) appliances are widely used in orthodontic treatment due to their ability to achieve controlled tooth movement and address various types of malocclusions. However, MB appliances represent plaque retention areas and make oral hygiene more difficult. As a result, patients undergoing fixed orthodontic treatment frequently experience considerable plaque accumulation during treatment, which may be associated with an elevated risk of increased gingival and plaque index values [1], gingival inflammation [2] or enamel demineralization [3, 4].

Standardised oral hygiene parameters, such as the Turesky modified Quigley and Hein Plaque Index [5] and the Rustogi modified Navy Plaque Index [6] are typically used to determine the amount of plaque present. This is usually done by applying a plaque disclosing agent to the teeth and visual categorisation of the stained areas. In the orthodontic context, plaque indices have been proposed that consider the specific plaque distribution around brackets, for example the Bonded Bracket Plaque Index or the Ortho Plaque Index [7]. However, the disadvantage of all index systems is that they are semi-quantitative and require complex calibration processes to ensure reproducible data.

Alternatively, planimetric quantification [8] can be used which describes plaque-covered areas as a percentage of the tooth surface area visible in the image. This allows for a continuous, objective, and reproducible assessment of plaque without relying on subjective scoring [9]. It was already shown that disclosed plaque can be captured with intraoral scanners as a 3D representation of the dentition and objectively quantified using planimetric methods [10]. In patients with MB appliances, planimetric plaque assessment has also been reported; however, previous approaches have predominantly relied on conventional photographic documentation [11]. For future applications and monitoring approaches, intraoral scanning could offer relevant advantages over photographs, particularly by enabling more consistent capture of posterior regions and improving access to oral surfaces, which are often difficult to document reliably using standard 2D photography.

However, highly reflective surfaces with ‘non-tooth’ shapes such as metal brackets can pose a problem for the algorithm used to create 3D objects from the 2D images captured during the scanning process. These artefacts may manifest as incomplete surface reconstructions, shadowing, or misclassification of plaque as part of the bracket or vice versa, which could impair the accuracy of digital plaque quantification.

This study aimed to evaluate the feasibility of imaging disclosed dental plaque using an intraoral scanner in individuals with MB appliances. Specifically, it investigated whether plaque on tooth surfaces can be reliably detected despite the presence of metal brackets.

## Patients, materials and methods

### Study design

This methodological study was approved by the Ethics Committee of the Medical Faculty of Medicine at the University of Marburg (ref. no. 23–220 BO) and registered in the German Clinical Trial Register (DRKS00033010).

The study was conducted in accordance with good clinical practice (ICH Harmonised Tripartite Guideline E6: Note for Guidance on Good Clinical Practice, CPMP/ICH/135/95 Step5) and the Declaration of Helsinki.

Sample size estimation was based on pilot data and calculated using MedCalc v20.251 (MedCalc Software Ltd., Ostend, Belgium). To evaluate the validity of plaque quantification with MB appliances in situ, the unit of analysis was the tooth surface. To ensure a representative range of plaque levels, the Ramfjord teeth (FDI 16, 21, 24, 36, 41, 44) [12] were analysed. A pilot study revealed a mean plaque coverage difference of −2.8 ± 6.8% before and after bracket debonding. The sample size calculation for Bland–Altman analysis (α = 0.05, β = 0.20, expected difference = 0, SD = 7, maximum allowable difference = 20) indicated a required sample size of 43 observations, corresponding to at least seven participants. To compare two bracket systems, 10 participants per group (total *n* = 20) were calculated. As only one visit was required, study completion by all participants was expected, without drop-outs.

### Patient recruitment

Participants in the study were adolescents and young adults with fixed MB appliances who were approaching the scheduled debonding stage of their orthodontic treatment. Recruitment was carried out through the standard information channels of the orthodontic department. Since bracket size may influence the evaluability of intraoral scans, only individuals with Mini Sprint^®^ II brackets (FORESTADENT Bernhard Förster GmbH, Pforzheim, Germany) or SPEED brackets (SPEED System™ Orthodontics, Strite Industries Ltd., Ontario, Canada) were included. Inclusion criteria were a minimum age of 14 years, written informed consent, and a complete permanent dentition (excluding third molars or teeth extracted for orthodontic reasons) with a closed dental arch. Furthermore, vestibular smooth surfaces had to be free of restorations extending beyond the extent of pit and fissure sealants.

Exclusion criteria were known intolerances to any of the materials used (Mira-2-Ton^®^ solution: sodium benzoate, potassium sorbate, C. 45410, C. 42090; OptraGate lip retractor: styrene-ethylene-butylene-styrene), dental deformities, spacing between teeth, and gingival recessions exceeding one-third of the root length. Individuals with physical or mental limitations of any kind that could interfere with the performance of the study procedures were also excluded.

### Procedure

The patients were recruited from the Department of Orthodontics. The study was performed between December 2023 and January 2025. Patients who were eligible for the study during their regular orthodontic treatment and who were interested in participating in the study received further information about the study, the additional time commitment, and the procedure. After written informed consent, the inclusion and exclusion criteria were assessed again. For participants under the age of 18, additional parental consent was obtained.

Twenty patients (15 females and 5 males) with a mean age of 17.0 ± 2.1 years were included in the study. Ten patients were treated with SPEED brackets, and ten with Mini Sprint^®^ II brackets. SPEED brackets are self-ligating, ultra-small brackets that do not require elastomeric ties, resulting in a more compact design. In contrast, Mini Sprint^®^ II brackets are conventional twin brackets (not self-ligating) with a small size, but larger than SPEED brackets.

At the following appointment (scheduled debonding session – T1), after removal of all elastics and archwires, but with the brackets still in situ, dental plaque was stained using the disclosing agent Mira-2-Ton^®^ solution (Hager & Werken GmbH & Co. KG, Duisburg, Germany). The solution was applied twice using a saturated foam pellet and rinsed with water for 10 s after each application.

Following plaque disclosing, a 3D-intraoral scan was performed using the Dexis IS 3800 W intraoral scanner (Dental Imaging Technologies Corporation, Charlotte, NC, USA). Scanning was carried out under standardized conditions. These included the use of a latex-free lip retractor (OptraGate; Ivoclar Vivadent, Schaan, Liechtenstein) to stabilise and retract the cheeks and lips during scanning. The scanned areas were kept as dry as possible using an air syringe and saliva ejector. The light on the dental unit was turned off during scanning to minimize reflections on smooth tooth surfaces. Each scan was consistently initiated in the lower jaw and then continued in the upper jaw to prevent washout effects, which primarily occur due to salivary pooling in the mandibular region.

After completion of the scans of the upper and lower jaws, the brackets were debonded as part of the patients’ ongoing orthodontic treatment, while the adhesive area remained in place. This procedure was independent of the study. Immediately afterwards (T2), plaque was disclosed again, and a second scan of the upper and lower jaws was performed using the same procedure as described above.

**Fig. 1:**
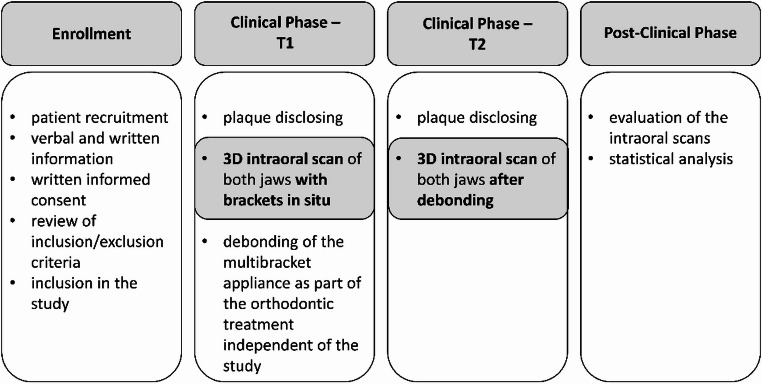
Flow chart

After completion of the clinical phase, the 3D-intraoral scans were analysed. The study design is illustrated in Fig. 1.

### Plaque assessment methods

Following the clinical phase of the study, the intraoral scans (.ply files) were processed using MeshLab software (Version 2022.02) [13]. For each participant, the two scans of the respective jaw (T1 and T2) were aligned by manually selecting at least 14 corresponding reference points per scan. After alignment, the paired scans were simultaneously rotated around the x, y, and z axes to achieve a standardized and optimal view of the tooth surface under examination. This ensured consistent visualization of both the mesial and distal aspects of the surface of interest.

Standardized screenshots were then generated, showing a frontal view of each Ramfjord tooth (FDI 16, 21, 24, 36, 41, 44), taken from the vestibular side at two time points: T1 (with brackets in situ) and T2 (after debonding). These images were saved as.jpg files for further analysis.

**Fig. 2:**
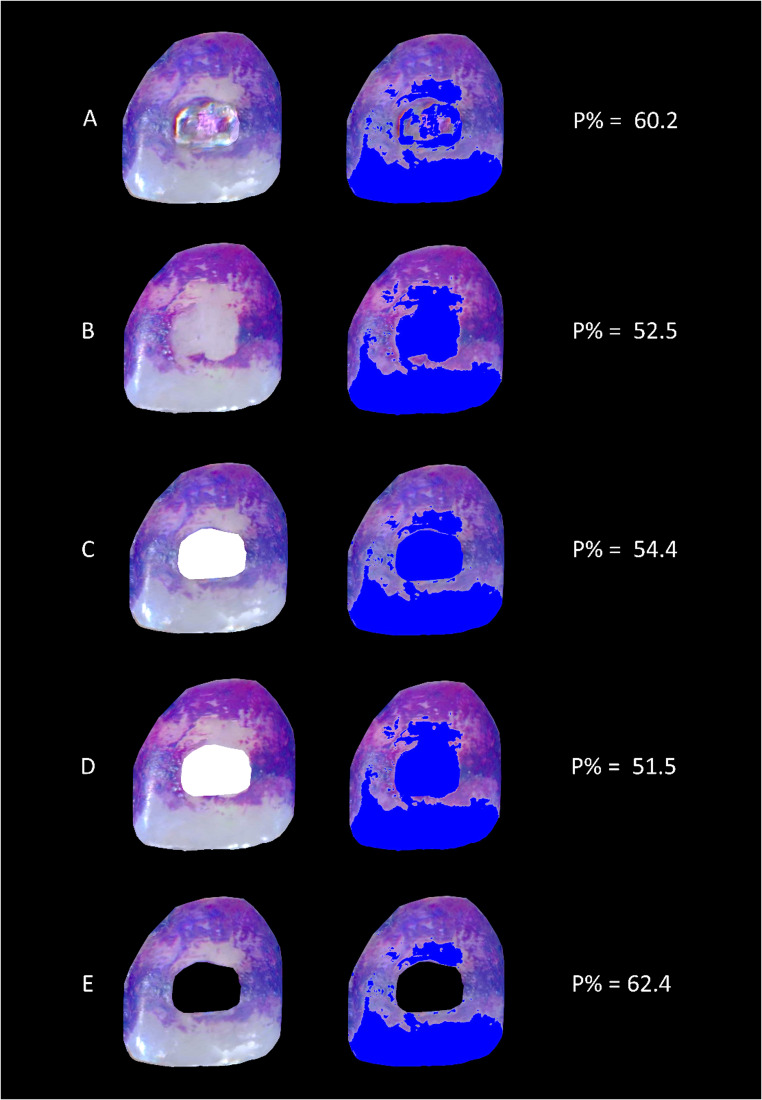
Representation of the examined conditions, left: cropped image from the intraoral scan, right: planimetric analysis. Blue areas indicate plaque-free tooth surfaces. (**A**) tooth with bracket: original image with bracket in situ. (**B**) tooth after debonding: image after debonding the bracket, but still the adhesive area on it. (**C**) tooth with bracket, masked: same as (**A**), but the area covered by the bracket was digitally masked and coloured in white. (**D**) tooth after debonding, masked: bracket-free image after debonding (same as in B), with the masked bracket area from (**C**) transferred onto it. (**E**) exposed surface only: the masked bracket area from image C was coloured black to exclude it from analysis, leaving only the exposed tooth surface for evaluation. The corresponding P% values are shown on the right

Subsequently, the images were processed using Adobe Photoshop (Photoshop CS5 extended, version 12.0 × 64, San Jose, California, USA). The relevant tooth surfaces were cropped and placed on a black background. The bracket areas in the T1 images were manually outlined and masked in two ways: once in white (representing plaque-free areas), and once in black (indicating surfaces not considered for plaque evaluation). This same bracket-shaped area was then transferred to the corresponding T2 image, positioned in exactly the same location on the bracket-free tooth surface. In total, five standardized images were created per tooth for planimetric analysis. A representative example is shown in Fig. 2:

These five images of each tooth were saved in .jpg format and used for planimetric plaque quantification. The analysis was conducted using a custom-written software tool based on the Julia programming language, utilizing the “ImageMagick” and “Images” packages (described in detail in [10]. Each .jpg image was converted into RGB format, and the red, green, and blue values of every pixel were extracted, transformed into a three-component vector, and stored separately. Two filtering steps were then applied to calculate the percentage of plaque coverage (P%): the first excluded all black background pixels (RGB = 0,0,0), and the second applied a defined threshold (RGB: = 1,0.25,1) to distinguish between plaque-covered and plaque-free areas. Batch processing enabled automated calculation and export of P% values. The output images were then visually compared with the original images, and the threshold applied to the green channel was adjusted in stepwise increments of 0.05 only in cases of substantial misclassification.

### Statistics

The Ramfjord teeth (FDI 16, 21, 24, 36, 41 and 44) were analysed at the tooth level (stratified by tooth group: molars, premolars, and incisors) to explore potential tooth-group–specific patterns and scanner-/anatomy-related effects. In addition, values from all six Ramfjord teeth were aggregated at the patient level to obtain a single value per patient, providing a clinically interpretable summary measure and reducing the impact of within-patient clustering (non-independence of tooth-level observations).

P% values were calculated for brackets in situ (with bracket) and after debonding the brackets (after debonding). Furthermore, P% values were calculated after masking the bracket area (with bracket, masked) and after transferring this mask to the post-debonding image (after debonding, masked). Finally, the images were evaluated excluding the bracket area (exposed surface).

The data were first checked for a sufficient Gaussian distribution (Kolmogorov-Smirnov-test). A significant deviation was only found for the exposed surface variable, which is therefore reported as the median [95% CI]. All other variables are expressed as mean ± standard deviation; parametric test procedures were applied except for exposed surface, where non-parametric procedures were used.

Differences in P% between conditions were tested with t-tests for dependent variables.

Agreement of P% values was analysed with Bland-Altman analyses, P% after debonding was set as reference. Bland-Altman analysis was used to evaluate whether two conditions yield comparable P% values in individual patients/teeth. Systematic bias describes the average difference, i.e., whether one condition tends to measure consistently higher or lower plaque coverage than the reference. Proportional bias indicates whether this difference becomes larger or smaller when overall plaque levels are high (or low), meaning that agreement may depend on the severity of plaque accumulation. The limits of agreement describe the range within which most individual differences lie, and therefore indicate how much two measurements may differ for a given patient/tooth in routine use.

Whether the P% values differed depending on the type of bracket as well as the tooth type was investigated using t-tests for independent samples except for P% on exposed surfaces, where Mann-Whitney-U-tests were applied.

### Reproducibility

Planimetric plaque quantification represents an objective and highly reproducible approach, as demonstrated in our previous work [9, 10]. In the present workflow, manual delineation of bracket areas was added, and the identical bracket-shaped mask was subsequently transferred to the corresponding T2 image and positioned at the same location on the bracket-free tooth surface. The alignment and preparation steps (standardized screenshots, cropping, and mask positioning on T2) were repeated by the same operator after a minimum interval of four weeks, whereas plaque quantification for the calibration dataset was performed independently by a second examiner. Agreement remained excellent in a calibration dataset comprising six participants with 12 teeth each (*n* = 72), yielding an ICC [95% confidence interval] of 0.972 [0.939–0.987] for manual bracket delineation and an ICC of 0.974 [0.935–0.991] for mask transfer to T2.

## Results

### Analysis on the patient level

All recruited patients completed the study. On the tooth surfaces around the brackets (exposed surfaces), a substantial plaque coverage of 45.9 P% [38.9; 51.0] was observed.

**Fig. 3:**
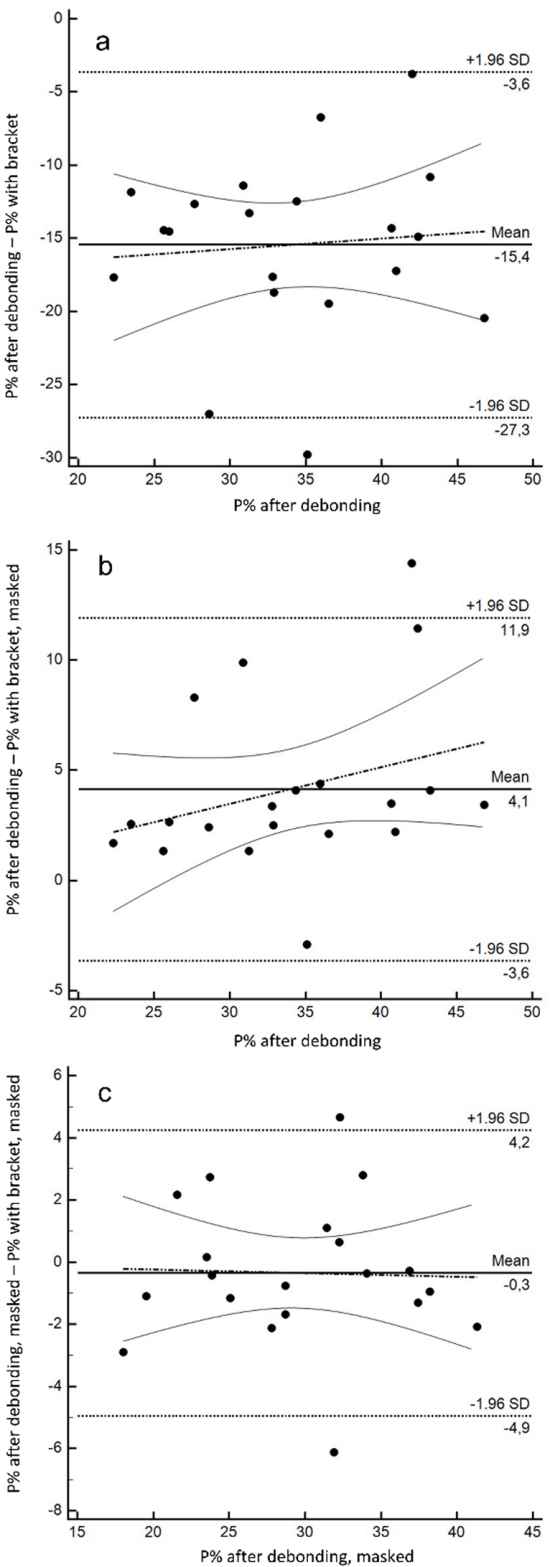
Bland-Altman analysis for the agreement of P% values: **a**: P% values with brackets compared to the reference (P% values after debonding), **b**: P% values with brackets, masked compared to the reference (P% values after debonding), **c**: P% values from images with brackets and after debonding, where the bracket area was masked on both; solid line: mean difference between the two methods (bias line), dashed line: mean difference ± 1.96 × SD (limits of agreement), broken line: regression line with 95% CI (solid lines)

Looking at the entire tooth surface, highest P% values were found with brackets in situ. Here, P% values were distinctly higher than after debonding (49.4 ± 8.9 vs. 34.0 ± 7.1; *p* <.001). Accordingly, the Bland-Altman analysis showed a distinct systematic (*p* <.001), but also a slight significant proportional bias (*p* <.001). P% values with brackets overestimated P% values after debonding somewhat less at higher overall plaque coverage than with lower plaque coverage values (Fig. 3a).

After masking the bracket (with bracket, masked), the P% values clearly approximated those after debonding, but still remained significantly lower (29.8 ± 7.0 vs. 34.0 ± 7.1; *p* <.001). The Bland-Altman analysis revealed a significant albeit small systematic (*p* <.001) but no significant (*p* =.732) proportional bias (Fig. 3b).

When the masked bracket area was also transferred to the images after debonding (after debonding, masked), both showed similar P% values (29.8 ± 7.0 vs. 29.5 ± 6.6; *p* =.518). Correspondingly, the Bland-Altman analysis showed neither a systematic (*p* =.517) nor a proportional (*p* =.892) bias as well as narrow limits of agreement (Fig. 3c).

### Analysis on the tooth level

The analysis at the tooth level considered both the two bracket systems as well as the different tooth types.

**Fig. 4:**
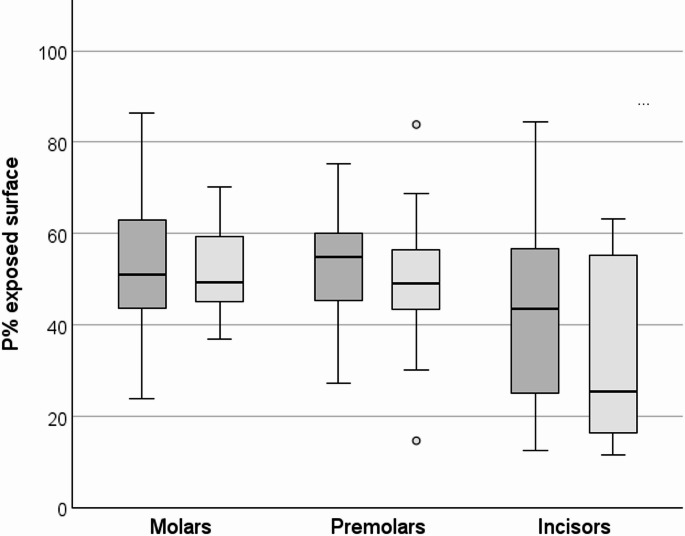
Boxplots of P% values on tooth surfaces excluding the bracket area (exposed surfaces) by tooth type. Dark grey: SPEED, light grey: Mini Sprint^®^ II

As the P% values aggregated by subject, the exposed tooth surfaces showed marked plaque coverage at the tooth level as well (Fig. 4). P% values for anterior teeth were lower overall than for molars and premolars (*p* <.01 each), which did not differ significantly (*p* =.965). Differences in P% values by bracket type did not reach significance for either molars and premolars or anterior teeth (*p* =.986, *p* =.301 and *p* =.142 resp.).

The P% values for the other parameters are displayed in Table 1. In general, no significant differences were found between the bracket systems. The only exception was P% values after debonding, which were slightly higher overall for SPEED than for Mini Sprint^®^ II (*p* =.037). When looking at the tooth types, however, this was only found for the anterior teeth (*p* =.040).

**Table 1:** P% values for the different variables overall and by tooth type (mean ± SD). Different upper-case characters indicate significant differences between bracket types

All	with bracket	after debonding	with bracket, masked	after debonding, masked
SPEED	52.5 ± 17.2^a^	36.5 ± 13.0^a^	31.4 ± 11.8^a^	31.4 ± 11.6^a^
Mini Sprint^®^ II	46.2 ± 17.8^a^	31.3 ± 13.6^b^	28.2 ± 11.8^a^	27.5 ± 11.9^a^
**Molars**				
SPEED	47.0 ± 15.4^a^	31.9 ± 8.0^a^	28.1 ± 6.3^a^	26.7 ± 5.9^a^
Mini Sprint^®^ II	48.6 ± 12.7^a^	30.5 ± 6.7^a^	27.8 ± 4.2^a^	26.0 ± 5.1^a^
**Premolars**				
SPEED	60.9 ± 13.6^a^	38.1 ± 10.6^a^	33.0 ± 8.6^a^	32.1 ± 8.3^a^
Mini Sprint^®^ II	53.1 ± 17.0^a^	35.7 ± 13.8^a^	31.7 ± 11.4^a^	31.0 ± 11.6^a^
**Incisors**				
SPEED	48.8 ± 19.6^a^	38.9 ± 17.4^a^	32.6 ± 17.1^a^	34.7 ± 16.3^a^
Mini Sprint^®^ II	37.4 ± 19.1^a^	27.5 ± 16.4^b^	25.0 ± 15.5^a^	25.1 ± 15.3^a^

**Fig. 5:**
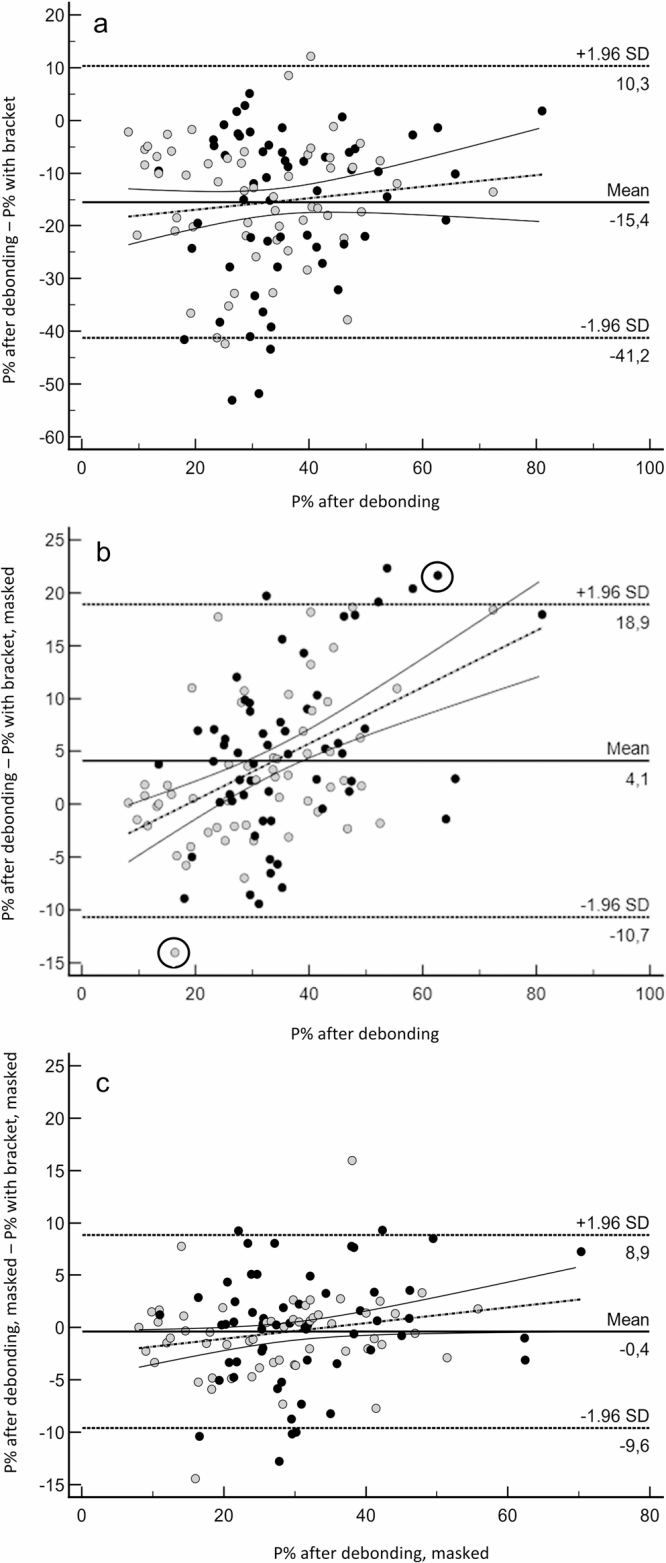
Bland-Altman analysis for the agreement of P% values: a: P% values with bracket to the reference (P% values after debonding), b: P% values with bracket, masked to the reference (P% values after debonding), c: P% values from images with brackets and after debonding, where the bracket area was masked on both. Black circles: SPEED brackets, grey circles Mini Sprint^®^ II; solid line: mean difference between the two methods (bias line), dotted line: mean difference ± 1.96 × SD (limits of agreement), broken line: regression line with 95% CI. The cases marked with a circle in (**b**) are shown in Fig. 6

In general, the Bland-Altman analysis revealed a distinct systematic bias for P% with bracket compared to P% after debonding (*p* <.001) but no proportional bias (*p* =.236) (Fig. 5a).

**Fig. 6:**
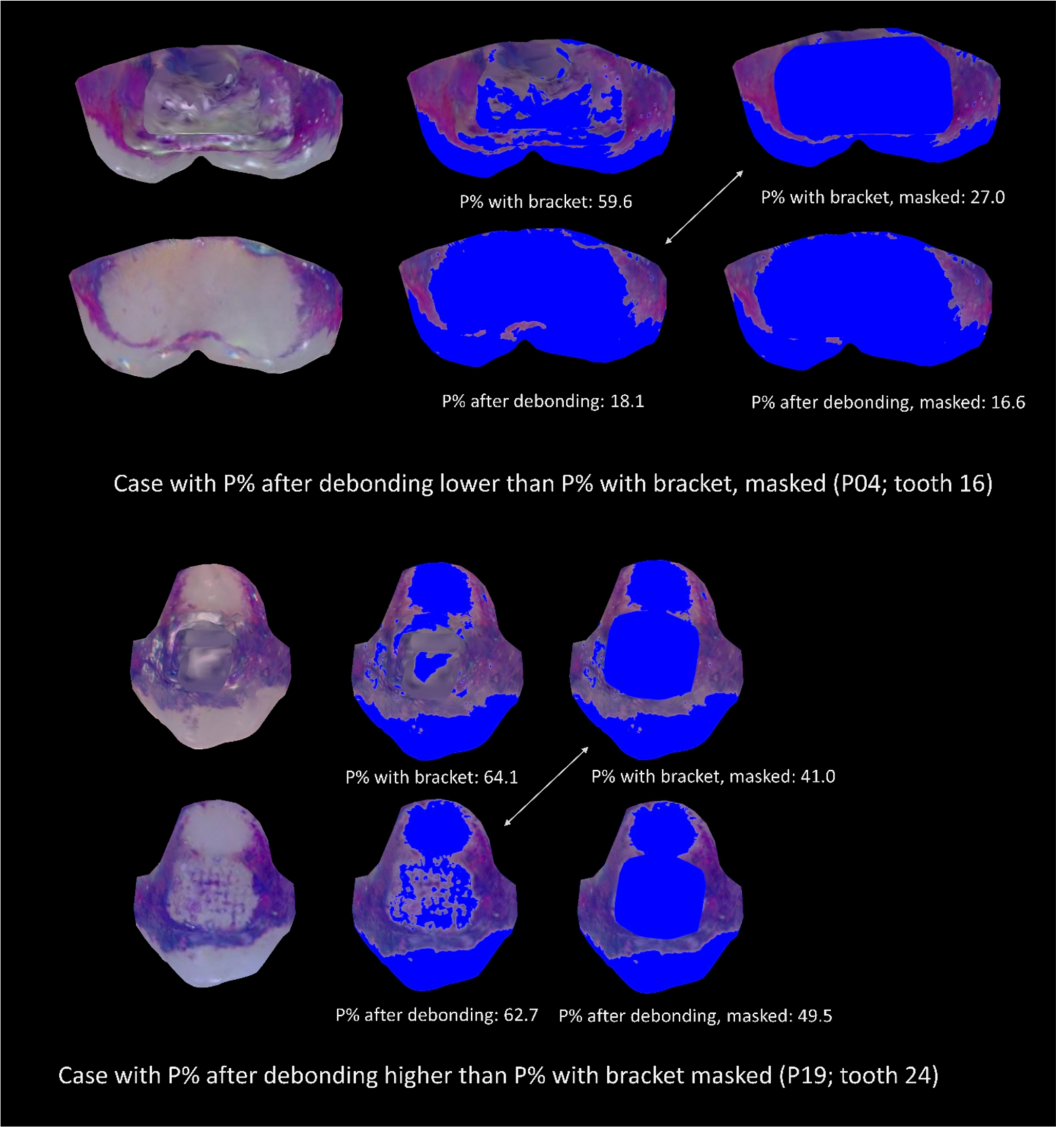
Cases representing the outliers marked in Fig. 5b. Top: the lower P% value on the image with the bracket area masked compared to P% after debonding can be explained by the blurred boundary of the gingival bracket area and the associated inaccuracy when masking the bracket area, or by plaque-covered bracket adhesive in the gingival area, which was removed when the bracket was detached. Bottom: the higher P% value on the image with masked bracket area compared to the P% after debonding is explained by retention of the revelator on grooves of the remaining bracket adhesive. Blue areas indicate plaque-free tooth surfaces

Comparing P% with bracket, masked with P% after debonding revealed a distinctly lower but still significant systematic bias (*p* <.001), however, a significant (*p* <.001) proportional bias was found (Fig. 5b). Two cases exceeding the limits of agreement are illustrated in Fig. 6. When the bracket areas were masked on both the image with brackets and after debonding, the systematic bias disappeared (*p* =.399), but a significant (*p* =.044) albeit small proportional bias remained (Fig. 5c).

Results from the Bland-Altman analyses as comparisons with the reference by bracket- and tooth type are given in Table 2. Comparisons showed a small and in part significant systematic bias, but with wide limits of agreement. Except for incisors, an additional proportional bias was evident.

**Table 2:** Bland-Altman analysis for the agreement of P% with bracket, masked to the reference (P% after debonding)

All	Mean difference [lower; upper limit of agreement]	*p* value systematic bias	Regression equation	*p* value proportional bias
SPEED	5.2 [−10.7; 21.1]	*p* <.001	y = −5.1 + 0.3 x	*p* <.001
Mini Sprint^®^ II	3.1 [−10.3; 16.5]	*p* =.001	y = −4.6 + 0.2 x	*p* <.001
**Molars**				
SPEED	3.8 [−10.0; 17.6]	*p* =.039	y = −14.5 + 0.6 x	*p* =.005
Mini Sprint^®^ II	2.7 [−10.1; 15.4]	*p* =.123	y = −21.1 + 0.8 x	*p* <.001
**Premolars**				
SPEED	5.1 [−15.2; 25.5]	*p* =.039	y = −19.7 + 0.7 x	*p* =.001
Mini Sprint^®^ II	4.0 [−10.8; 18.8]	*p* =.030	y = −7.1 + 0.3 x	*p* =.009
**Incisors**				
SPEED	6.3 [−6.3; 19.0]	*p* <.001	y = 3.0 + 0.1 x	*p* =.327
Mini Sprint^®^ II	2.5 [−10.4; 15.4]	*p* =.106	y = −1.3 + 0.1 x	*p* =.137

## Discussion

Orthodontic appliances add artificial surfaces and markedly increase biofilm-retentive sites, which promotes greater plaque accumulation and may facilitate a shift towards a more cariogenic and periodontopathogenic microbial composition [14]. In addition, fixed appliances can reduce natural self-cleansing and impair mechanical biofilm removal during toothbrushing [15].

These conditions underline the need for regular, close-meshed oral hygiene instruction and motivation in patients with MB to reduce caries risk during treatment. However, achieving adequate home-care adherence, particularly in the typical orthodontic age group, remains challenging, emphasizing the need of the development of practical, patient-centred strategies to improve daily oral hygiene.

Plaque disclosing agents are a well-established tool in this context, as they make biofilm visible and thereby support both patient education and clinical assessment. Disclosed plaque helps to identify insufficiently cleaned areas, enables individualized instruction, and can enhance motivation; for example, visually supported interventions have been shown to improve oral hygiene in patients with fixed appliances [16]. Intraoral scanners could further strengthen such visually guided approaches by providing a manipulable 3D representation of the dentition and facilitating visualization of hard-to-assess regions (e.g., molars and lingual surfaces).

The present study demonstrates that planimetric plaque quantification on intraoral scans can be applied even in the presence of MB. As expected, patients with MB exhibited considerably high levels of plaque accumulation. The percentage of plaque-covered surface (P%) was 45.9% [38.9; 51.0] when only the exposed tooth surface was considered, and 49.4% ± 8.9% when the entire tooth surface including the brackets was analysed. By comparison, young adults without MB appliances showed a P% of 24.6% ± 8.0% after 72 h without oral hygiene, and 13.8% ± 4.6% directly after toothbrushing [9].

The choice of evaluation method had a significant influence on the assessment of plaque accumulation on teeth undergoing orthodontic treatment. If the entire tooth surface is included in the analysis, together with the bracket in situ, this inevitably means that plaque on the bracket is also recorded. Consequently, this evaluation method resulted in the highest P% values (49.4% ± 8.9%). However, plaque on bracket surfaces appeared more diffuse than on natural tooth structures. In some images, metallic surfaces were partially misclassified as plaque-covered, while in others, the distinction was clear posing rendering planimetric analysis challenging.

Following bracket debonding, P% decreased distinctly. This condition also served as a reference, as interference from metallic surfaces could be excluded, and any additional impact on plaque accumulation from manipulation of residual bonding material was avoided. Nevertheless, the measured value was still 12% higher than that of the variant with the bracket area masked. This result is probably due to dye retention in the remaining surface irregularities of the composite residues as shown in Fig. 6. This is particularly evident for the anterior teeth.

If the bracket areas were masked of both the data sets with brackets and after debonding, consistent and well-matched plaque quantifications were obtained. This finding demonstrates that the intraoral scanner used here is capable of reliably detecting and visualising disclosed plaque in regions immediately adjacent to orthodontic brackets. This suggests that the presence of brackets does not significantly interfere with the scanner’s ability to capture biofilm in surrounding regions, thereby validating its utility for quantitative plaque assessment even when fixed appliances remain in situ.

Masking the bracket area in white, thus marking it “plaque-free” for our planimetric evaluation software, was methodologically necessary to enable comparison with the after bracket debonding condition. This approach, however, still counted the bracket area in the planimetric evaluation. Clinically, the relevant outcome is plaque coverage on the tooth surfaces outside the bracket. When the bracket area was instead masked black, our filter settings excluded it from tooth-surface evaluation, reducing the analysed area and thereby increasing P% to 45.9 [38.9; 51.0].

Given that bracket types vary in size and thereby in the bracket-to-tooth size ratio shaping the bonded interface we expected tooth-type–specific effects. Yet across all analyses, bracket type had no measurable impact. Although anterior teeth showed a statistically significant difference after debonding, the effect was small and unlikely to matter clinically, it disappeared once the bracket area was masked and was absent in analyses with brackets in place.

One limitation of this study is that investigations were restricted to Ramfjord teeth. Although these are widely regarded as representative of the entire dentition [17–20], they may not fully capture the variability of plaque distribution across all teeth. Moreover, only one scanner type was used. A further limitation is that only two metal bracket systems were analysed. Tooth-coloured brackets (ceramic/composite) were not included. Their colour and translucency may reduce contrast with disclosed plaque and enamel, and differences in bracket geometry could affect segmentation. Other bracket designs, lingual bonded brackets and ligation methods may also alter plaque accumulation patterns and scanner artefacts. To improve generalizability, future studies should include multiple scanning technologies and a broader range of bracket types, especially tooth-coloured systems with varying base sizes and designs. Furthermore, automation of the plaque quantification workflow would be an important next step to enable broader implementation beyond research and facilitate its use in everyday clinical practice.

In conclusion, planimetric quantification of dental plaque using intraoral scans proves to be a valid and practical method, even in the presence of fixed orthodontic appliances, and it opens up further interesting research avenues in the context of multibracket treatment. Depending on the research question, it is advisable to scan the entire dental arch after removal of the archwire and excluding the bracket areas from plaque analysis by masking them. Both approaches allow for an objective and standardised evaluation of plaque distribution under real clinical conditions, thereby contributing meaningfully to the development of targeted preventive strategies in orthodontic care. Although this proof-of-concept study demonstrates technical feasibility, the clinical applicability and utility of intraoral-scan–based planimetric plaque quantification in routine orthodontic practice remain to be established.

## Data Availability

The data is available from the corresponding author on reasonable request.
